# Self-Inhibitory Activity of Trichoderma Soluble Metabolites and Their Antifungal Effects on *Fusarium oxysporum*

**DOI:** 10.3390/jof6030176

**Published:** 2020-09-17

**Authors:** Samuel Álvarez-García, Sara Mayo-Prieto, Santiago Gutiérrez, Pedro Antonio Casquero

**Affiliations:** 1 Grupo Universitario de Investigación en Ingeniería y Agricultura Sostenible (GUIIAS), Instituto de Medio Ambiente, Recursos Naturales y Biodiversidad, Universidad de León, Avenida Portugal 41, 24071 León, Spain; smayp@unileon.es (S.M.-P.); pacasl@unileon.es (P.A.C.); 2 Grupo Universitario de Investigación en Ingeniería y Agricultura Sostenible (GUIIAS), Área de Microbiología, Escuela de Ingeniería Agraria y Forestal, Universidad de León, Campus de Ponferrada, Avenida Astorga s/n, 24401 Ponferrada, Spain; s.gutierrez@unileon.es

**Keywords:** self-inhibition, autotoxicity, autoinhibition, membrane assay, secondary metabolites, diffusible metabolites, antifungal, fungi, Trichoderma, Fusarium

## Abstract

Self-inhibitory processes are a common feature shared by different organisms. One of the main mechanisms involved in these interactions regarding microorganisms is the release of toxic diffusible substances into the environment. These metabolites can exert both antimicrobial effects against other organisms as well as self-inhibitory ones. The in vitro evaluation of these effects against other organisms has been widely used to identify potential biocontrol agents against phytopathogenic microorganisms. In the present study, we performed membrane assays to compare the self-inhibitory effects of soluble metabolites produced by several Trichoderma isolates and their antifungal activity against a phytopathogenic strain of *Fusarium oxysporum*. The results demonstrated that *Trichoderma* spp. present a high self-inhibitory activity in vitro, being affected in both their growth rate and the macroscopic structure of their colonies. These effects were highly similar to those exerted against *F. oxysporum* in the same conditions, showing no significant differences in most cases. Consequently, membrane assays may not be very informative by themselves to assess putative biocontrol capabilities. Therefore, different methods, or a combination of antifungal and self-inhibitory experiments, could be a better approach to evaluate the potential biocontrol activity of microbial strains in order to pre-select them for further in vivo trials.

## 1. Introduction

Autotoxicity and self-inhibition have been described as common traits shown by different species, genera, and kingdoms [1]. Some general mechanisms have been proposed to explain these processes, mainly the accumulation and release of different toxic compounds to the environment, and also the self-inhibitory activity of extracellular self-DNA or conspecific DNA [1]. However, competition for resources, quorum sensing, and reproductive issues have been pointed out as the most likely and major ecological factors that motivate this behavior [2].

Regarding filamentous fungi, some studies have been conducted to evaluate self-inhibitory processes, allowing the identification of different autotoxic compounds [3,4]. Nonetheless, most of these studies are focused on the concentration-dependent self-inhibition of fungal spore germination [3,4,5]. Moreover, autotoxicity has been proposed as a way to drive directional growth, accounting for the characteristic radial expansion of fungal colonies [6]. As for the genus Trichoderma, there are very few references to self-inhibition. In this regard, most significant studies point to the self-inhibitory effects of *Trichoderma harzianum* mediated by extracellular self-DNA [1]. Besides, to our knowledge, there are no previous studies comparing self-inhibitory and antifungal effects produced in vitro by *Trichoderma* spp. or any other biocontrol agent. Trichoderma is a ubiquitous genus of filamentous fungi, widely used as a biocontrol agent against many phytopathogenic microorganisms [7,8], as well as for crop biostimulation [9,10] and as a general model to sustain crop productivity [11].

On the other hand, *Fusarium oxysporum* is a common endophyte and phytopathogenic filamentous fungus that thrives in numerous important crops [12], *F. oxysporum* f. sp. *phaseoli* being one of the forms that mainly affects bean plants (*Phaseolus vulgaris* L.) [13]. Furthermore, many studies highlight the antifungal activity of soluble metabolites [8,14,15], as well as volatile ones [5,14,16], produced by *Trichoderma* spp. against different Fusarium species.

The hypothesis of this work is that soluble metabolites released in vitro by *Trichoderma* spp. produce both self-inhibitory effects and antifungal effects against *F. oxysporum*. The main objective of the present study is to evaluate the self-inhibitory effects of soluble metabolites produced by several Trichoderma isolates and to compare them to the antifungal activity exerted by the same fungal strains against a phytopathogenic strain of *F. oxysporum*.

## 2. Materials and Methods

### 2.1. Microbial Strains and Culture Conditions

Ten Trichoderma strains were used to evaluate the activity of their diffusible metabolites: seven *T. harzianum*, one *T. citrinoviride*, one *T. velutinum*, and one *T. gamsii*. Out of the ten strains, eight of them were isolated from bean (*P. vulgaris* L.) fields belonging to de Protected Geographical Indication (PGI) “Alubia de La Bañeza-León”, while the other two were isolated from sugar beet crops growing in the same area [17]. Trichoderma strains were selected in such a way that at least either the fungal species, the place of origin, the bean variety (or crop) they came from, or the source they were isolated from (seed or soil) differed among them (Table 1).

A strain of *F. oxysporum* (F3 from now onwards) was isolated from bean (*P. vulgaris*) fields belonging to the same PGI and was selected for its high virulence against this crop.

All fungal strains were preserved in 50% glycerol spore suspension at −80 °C and stored in the “Pathogens and Antagonists Collection” at the “Pest and Diseases Diagnosis Laboratory” (PALDPD, University of León, León, Spain). All cultures were activated on PDA (Sigma Aldrich, St. Louis, MO, USA) at 25 °C.

### 2.2. In Vitro Evaluation of Antifungal and Self-Inhibitory Activity of Soluble Metabolites Produced by Trichoderma spp.

**Figure 1:**
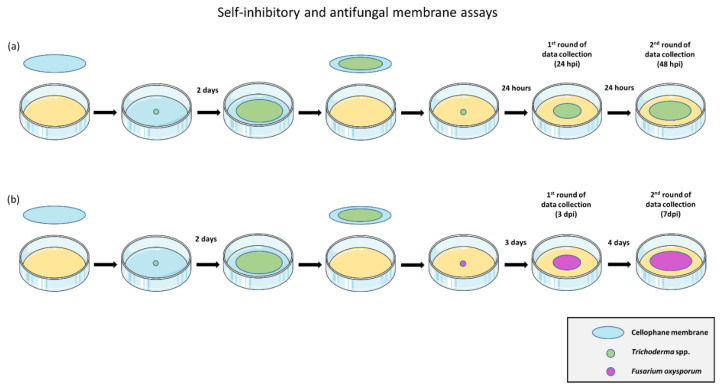
Self-inhibitory and antifungal membrane assays. (**a**) Evaluation of the effects of *Trichoderma* spp. diffusible metabolites on the same Trichoderma strain. (**b**) Evaluation of the effects of *Trichoderma* spp. diffusible metabolites on *F. oxysporum* F3.

A membrane assay was performed, as described by Mayo et al. (2015) [7], by placing a sterile cellophane membrane to cover the potato-dextrose-agar (PDA, Sigma Aldrich, St. Louis, MO, USA) medium in 90 mm Petri dishes (Sigma-Aldrich Chemie GmbH, Steinheim, Germany). A 6 mm plug from the edge of a 2 day-old Trichoderma culture was placed in the center of each plate over the cellophane and was grown for 2 days, letting the soluble metabolites diffuse to the medium, yet avoiding its colonization by the fungal mycelium. After that, membrane and fungi were removed, and a 6 mm mycelial plug from the edge of the 2 day-old culture of the same Trichoderma strain was placed in the center of the dish. Controls were performed in the same manner but without growing Trichoderma over the membranes prior to the inoculation. Diameters of the colonies were measured in three different directions with a ruler to the farthest edge of mycelial growth (via visual detection of the most outer hyphal apex) 24 and 48 h post-inoculation (hpi) (Figure 1a). The diameter of each replicate was considered as the mean of its three measurements. The second round of data collection was determined to be 48 hpi, taking into account that the fastest-growing Trichoderma strains reached the edge of the plate at this time.

The inhibitory activity of Trichoderma metabolites on *F. oxysporum* was performed as described above, but inoculating a 6 mm plug from the edge of a 5 day-old *F. oxysporum* culture on the center of the plates after removing the membranes. The diameters of the colonies were measured in the same way, but 3 and 7 days post-inoculation (dpi) (Figure 1b), as the slower mycelial growth of *F. oxysporum* controls reached the same diameter after 3 and 7 days as Trichoderma controls after 24 and 48 h. The second round of data collection was determined to be 7 dpi, taking into account that F3 colonies reached the edge of the plate at this time. From now onwards, measures taken 24 hpi for *Trichoderma* spp. and 3 dpi for F3 will be referred to as the first round of data collection, while 48 hpi for *Trichoderma* spp. and 7 dpi for F3 will be considered to be the second round of data collection, in order to compare self and heterologous inhibition.

Controls were performed in the same manner but without growing *Trichoderma* spp. over the cellophane membrane. The 1st round of data collection was performed when fungal colonies in the controls reached 1/3 of the plate (24 hpi for *Trichoderma* spp. and 3 dpi for *F. oxysporum*). The 2nd round of data collection was performed when fungal colonies in the controls reached the edge of the plate (48 hpi for the fastest *Trichoderma* spp. and 7 dpi for *F. oxysporum*). Diameters were measured with a ruler in three different directions. Four replicates per treatment were performed. All cultures were performed on PDA (Sigma Aldrich, St. Louis, MO, USA) at 25 °C.

### 2.3. Data Treatment and Statistical Analysis

**Figure 2:**
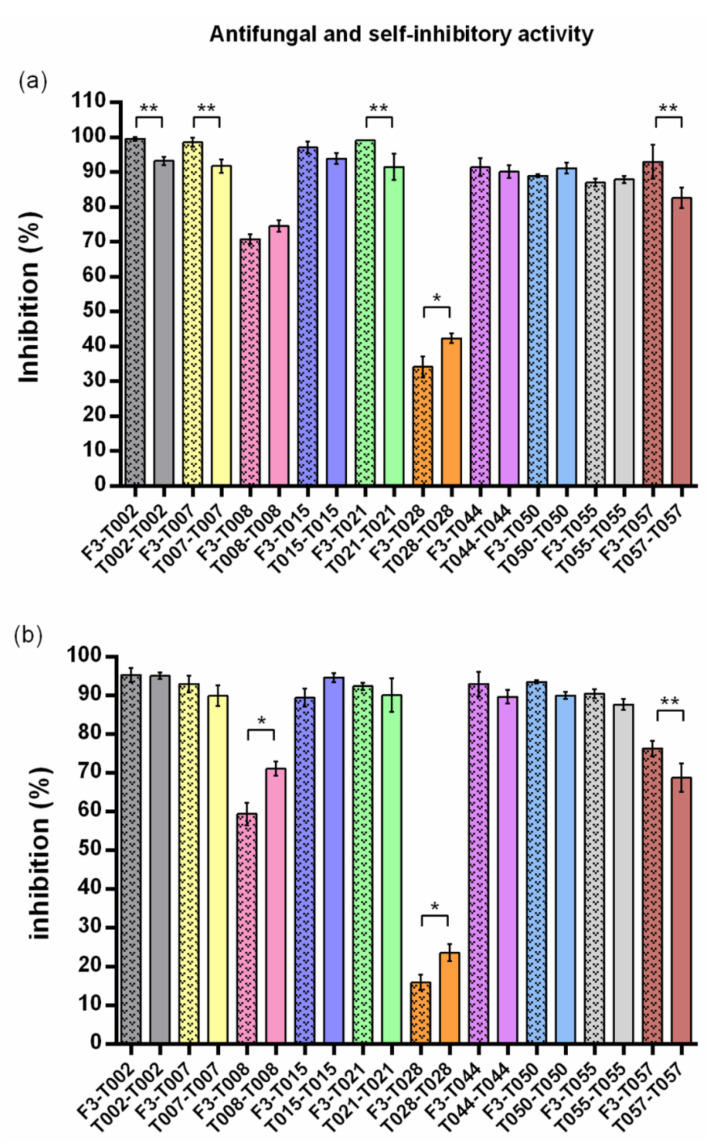
Self-inhibitory activity of the soluble metabolites from the *Trichoderma* spp. evaluated (plain columns), and their antifungal activity against *F. oxysporum* F3 (shadowed columns). (**a**) First round of data collection (24 hpi for Trichoderma and 3 dpi for F3); (**b**) Second round of data collection (48 hpi for Trichoderma and 7 dpi for F3). Columns indicate the mean of PI values (%) and their standard deviation (SD). Each color represents a treatment with a different Trichoderma strain. Shadowed columns represent PI values produced by Trichoderma strains against F3, while plain ones represent self-inhibitory PI values produced by each Trichoderma strain on itself. (*) indicate significantly higher antifungal than self-inhibitory activity (*p* ≤ 0.05) within the same Trichoderma isolate. (**) indicate significantly higher self-inhibitory than antifungal activity (*p* ≤ 0.05) within the same Trichoderma isolate. No asterisks mean no differences between self-inhibitory and antifungal activity (*p* ≤ 0.05). PIs were estimated in relation to the control using the following equation: PI (%) = [(C − T/C) × 100] [16]. A one-way analysis of variance (ANOVA, *p* ≤ 0.05) was performed, and differences were estimated using Tukey’s post hoc test (*p* ≤ 0.05) to compare the self-inhibition and antifungal effects for each Trichoderma strain.

Microbial growth was considered as the mean diameter of the three measures from each replicate, and 6 mm were subtracted from all measures to avoid distortions produced in the percentage of inhibition (PI) by the diameter of the plugs, as shown by Mutawila et al. (2016) [15]. PIs were estimated in relation to the control using the following equation: PI (%) = [(C − T/C) × 100] [16], where C is the diameter of the control and T is that of each Trichoderma treatment. PIs were analyzed with one-way analysis of variance (ANOVA, *p* ≤ 0.05) after confirmation of normality and equality of variances. Subsequently, treatments were contrasted between them and with their controls using Tukey’s post hoc test (*p* ≤ 0.05). Statistical analyses were performed separately for self-inhibition and antifungal activity of the ten *Trichoderma* spp. (Table 2), as well as to compare self-inhibition and antifungal activity of each Trichoderma isolate (Figure 2). Four replicates were performed for each treatment.

## 3. Results and Discussion

Under the referred conditions, all Trichoderma isolates showed important antifungal effects on F3 growth (Table 2). *T. harzianum* isolates consistently demonstrated a higher inhibitory activity than the other three Trichoderma species, with PIs ranging from 99.51% to 86.92% at day 3 post-inoculation, and from 95.25% to 89.40% in day 7 post-inoculation. In contrast, *T. citrinoviride* T008 showed lower PI values (70.73% in day 3 and 59.38% in day 7), while *T. velutinum* T028 exerted the lowest inhibition of all strains, with 34.15% in day 3 and a mere 15.89% in day 7. Finally, *T. gamsii* T057 showed similar PI to those of some *T. harzianum* in day 3 (92.90%) with a significant decrease in day 7 (76.27%). All treatments showed significant differences (*p* ≤ 0.05) compared to the growth of F3 controls. These results are in accordance with previous studies regarding the antifungal activity of *Trichoderma* spp. on *F. oxysporum* [18,19].

Interestingly, the self-inhibitory effects of the tested Trichoderma strains seem to be highly similar to the abovementioned antifungal inhibitory ones on F3. In this regard, five strains (T008, T015, T044, T050, and T055) showed no significant differences between self-inhibitory and antifungal activities during the first round of data collection (Figure 2a). Moreover, this number amounted to seven strains (all *T. harzianum* tested) in the second round of data collection (Figure 2b). The remaining three Trichoderma strains (T008, T028 and T057) showed, however, differences between self and heterologous inhibition, although following a similar trend. Their IPs for both heterologous antifungal and self-inhibitory effects were significantly lower (*p* ≤ 0.05) than those of the *T. harzianum* strains (Table 2). Self-inhibitory PIs of *T. harzianum* strains were very high and ranged from 95.04% (T002, 48 hpi) to 87.66% (T055, 48 hpi). However, T008, T028, and T057 showed, respectively, self-inhibitory PIs of 74.51%, 42.29%, and 82.53% after 24 h, and 71.02%, 23.57% and 68.74% after 48 h (Table 2). Additionally, T008 and T028 were the only two strains showing significantly higher self-inhibition than antifungal inhibition against F3. Other strains showed slightly higher self-inhibitory effects than their antifungal ones, but were not statistically significant (*p* ≤ 0.05) (Figure 2).

These results seem to indicate that the antifungal and self-inhibitory effects exerted in vitro by *Trichoderma* spp. are closely related and might be general traits shared for both heterologous competition with other fungal species in the environment, as well as ecological adaptations within the same genus, species, and strain. The latter likely being related to directional centrifugal growth of filamentous fungi [6], competition for resources, and/or reproductive processes [3]. The self-inhibitory activity presented here seems to be a non-specific one, and therefore, different from that proposed by Mazzoleni et al. (2015) in *T. harzianum*, mediated by conspecific DNA [1]. These heterologous and autotoxic effects may be related to those observed in fairy-ring-forming basidiomycetes, which have been proposed as one of the mechanisms behind the formation of their characteristic growth rings in the soil and their important influence on the ecosystemic scale [20]. The highly similar effects observed between F3 inhibition and Trichoderma self-inhibition may be partly due to their relative phylogenetic and ecological proximity, being both soilborne filamentous ascomycetes related to the rhizosphere and plants, as epiphytic or endophytic fungi. New studies are needed to elucidate whether these similar effects are also found when comparing other filamentous fungi and less related microorganisms.

The results also point out that self-inhibition varies between Trichoderma strains and species, suggesting that either they produce higher quantity and/or stronger autotoxic metabolites, or they possess different detoxification capabilities to confront these compounds. We propose that, taking into account the similarities presented here between self and heterologous antifungal inhibitory effects within the same strains, the differences seen between strains are mostly due to different antifungal activity and/or the amount rather than different detoxification capabilities. Besides, the high PI values, especially regarding those referred to self-inhibition, are likely due to an extremely high accumulation of soluble metabolites in the PDA medium, derived from the unnatural conditions of the in vitro design. Nevertheless, with the available data, it is not possible to determine whether the observed effects are produced by toxic subproducts of fungal metabolism or secondary metabolites that are purposefully released for antifungal activity. It also is not possible to assure that both the self-inhibitory and the heterologous antifungal activities are produced by the same metabolites, even though the very similar PI values between them seem to point in this direction. New studies should be directed to elucidate these aspects.

**Figure 3:**
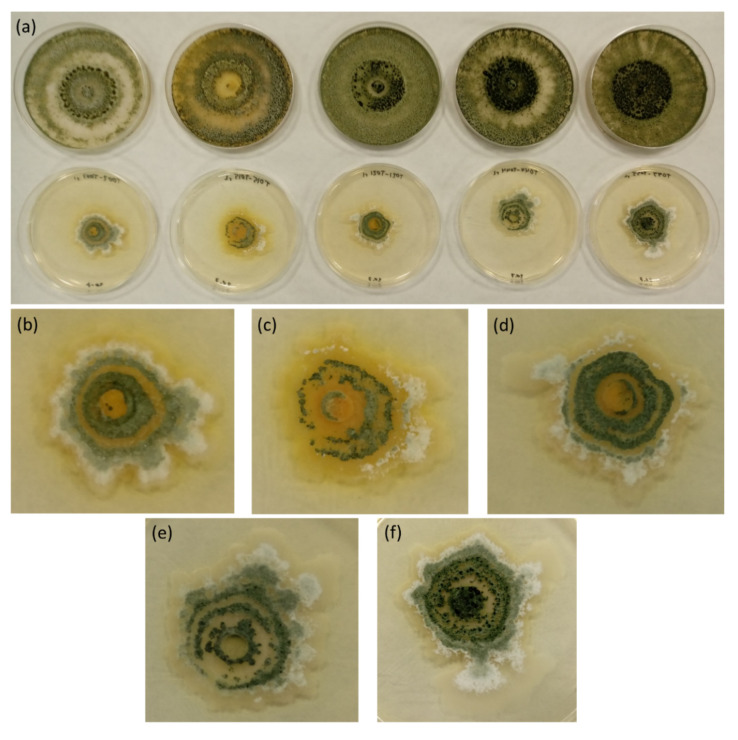
Development of some Trichoderma colonies after 7 days growing on PDA medium with soluble metabolites produced by the strain itself. Aberrant arbuscular shapes can be seen in the treatments. (**a**) Controls without metabolites (upper row; left to right: T002, T015, T021, T044, and T055); Trichoderma treatments (lower row; left to right: T002, T015, T021, T044, and T055), (**b**) T002 detail, (**c**) T015 detail, (**d**) T021 detail, (**e**) T044 detail, and (**f**) T055 detail.

In addition, as can be seen in Figure 3, Trichoderma colonies suffered a clearly aberrant growth when exposed to their own diffusible metabolites, showing uncharacteristic arbuscular shapes instead of their common circular colonies with homogeneous edges, clearly observed seven days after inoculation. This aberrant growth in the macroscopic level may indicate microscopic, anatomical abnormalities in the fungal hyphae, as well as physiological alterations [21,22]. Further microscopic and molecular investigations are needed to unveil these anatomical and physiological traits.

It is well known that in vitro assays are only an approximation of more complex natural contexts [23] and that, at least in the field of biological control and microbial interactions, they always need to be completed and validated by subsequent in vivo trials [7,17]. Nevertheless, the overall results here presented pose an additional doubt on membrane assays, and perhaps, on other in vitro related methods used to evaluate fungal soluble metabolites. These experiments are widely used as preliminary studies to help identify new bioactive compounds or microbiological strains with potential biocontrol capabilities [23,24,25,26]. However, our findings suggest that membrane assays, at least in the reported conditions, do not seem to be a very informative in vitro indicator of the real biocontrol activity that a fungal strain may exert in natural conditions. Therefore, these assays would still be very useful to identify bioactive compounds, but not so much to select putative biocontrol strains. In this regard, taking into account that most Trichoderma strains tested in the present study showed no significant differences between their self-inhibitory activity and their antifungal activity against *F. oxysporum*, we believe that their high in vitro inhibitory activity alone is not enough to claim a potential biocontrol activity, being likely biased by the unnatural concentrations reached in the plates. Thus, different experimental laboratory designs could be more adequate to evaluate these traits before transitioning to in vivo and field trials. For example, Kron et al. (2020) [27] demonstrated that ex vivo assays using *Pseudomonas orientalis* mutants against *Erwinia amylovora* were more informative than in vitro assays.

In addition, we suggest that if membrane assays are used to identify potential biocontrol strains, autotoxic studies, as the here presented using the same strains, should be conducted in identical conditions in order to compare and select those microbial agents showing both high antimicrobial activity alongside a lower self-inhibitory one. For example, in our case, while T002, T007, T015, and T021 show very high antifungal effects on the first round of data collection, we may consider discarding T015 as a potential biocontrol strain, as its antifungal activity against F3 is not significantly different from its own self-inhibitory one (Figure 2a). However, during the second round of data collection, T057 was the only strain showing significantly higher heterologous than self-inhibitory effects (Figure 2b). Thus, even though it had a lower antifungal activity than other strains, it might be adequate to select it for further in planta or in vivo experiments. This is further supported by the fact that T057 strain showed the highest difference between antifungal and self-inhibitory activities during the first round (Figure 2a). However, the validity of these hypotheses and proposals need to be tested by future plant and field trials, as well as by using a wider variety of both biocontrol agents and phytopathogenic strains to assess the presence of the same traits in other microorganisms.

Finally, a deeper understanding of self-inhibitory mechanisms within biocontrol strains, species, and genera might be a key factor to develop commercial pesticides based on a mixture of similar biological control agents. Additionally, this knowledge would be very useful to determine the ideal concentration of the agent when applying it on the field in order to avoid self-inhibitory effects, as well as to select the adequate growth conditions for its industrial production.

## 4. Conclusions

To summarize, the results presented demonstrate that the majority of the tested *Trichoderma* spp. show, in vitro, a high self-inhibitory activity mediated by secreted soluble metabolites. This activity varied among different strains and species, with *T. harzianum* showing the highest self-inhibitory effects. Trichoderma strains are affected both in their growth rate and the macroscopic structure of their colonies, arguably reflecting microscopic structural and physiological abnormalities in the fungal hyphae. These growth-inhibitory effects are highly similar to those exerted against *Fusarium oxysporum* in the same conditions, especially for *T. harzianum* isolates, indicating a likely comparable mechanism in both cases. In this regard, the great inhibition observed in both cases for most Trichoderma strains is possibly derived from the extremely high concentration of metabolites accumulated in the culture medium. As a result, membrane assays seem to not be very informative by themselves, in regards the direct biocontrol capabilities of the tested strains. Therefore, different methods or a combination of antifungal and self-inhibitory experiments could be a better approach to preliminarily evaluate the potential biocontrol activity of microbial strains in order to pre-select them in vitro for further in vivo trials. This strategy may serve to optimize the process of pre-selection, helping to save time and resources in the subsequent phases of the research. Nevertheless, these conclusions need to be further investigated and confirmed by new assays, involving both in vivo studies and a wider variety of biocontrol and phytopathogenic microbial strains.

## Figures and Tables

**Table 1:** Code, species, source, and area of origin of the *Trichoderma* isolates used in the assays.

Code	*Trichoderma* Species	Crop	Source	Municipality	Area
**T002**	*T. harzianum*	Riñón menudo (bean)	seed	Moscas del Páramo	El Páramo
**T007**	*T. harzianum*	Pinta (bean)	seed	Sueros de Cepeda	Astorga
**T008**	*T. citrinoviride*	Pinta (bean)	seed	Fresno de la Vega	Esla-Campos
**T015**	*T. harzianum*	Riñón menudo (bean)	seed	Veguellina de Fondo	El Páramo
**T021**	*T. harzianum*	Pinta (bean)	seed	Altobar de la Encomienda	El Páramo
**T028**	*T. velutinum*	Riñón (bean)	soil	Otero de Escarpizo	Astorga
**T044**	*T. harzianum*	Riñón (bean)	soil	Javares de los Oteros	Esla-Campos
**T050**	*T. harzianum*	Canela (bean)	soil	Bercianos del Páramo	El Páramo
**T055**	*T. harzianum*	Sugarbeet	soil	La Milla del Páramo	El Páramo
**T057**	*T. gamsii*	Sugarbeet	soil	La Milla del Páramo	El Páramo

**Table 2:** PI values (%) of self-inhibition and F3 inhibition produced by Trichoderma isolates. Results are expressed as the mean ± standard deviation (SD) of PI (%) from the four replicates. First round of data collection (24 hpi for *Trichoderma* spp. and 3 dpi for F3), and Second round of data collection (48 hpi for *Trichoderma* spp. and 7 dpi for F3).

		First Round of Data Collection				Second Round of Data Collection			
Code	*Trichoderma*	Self-Inhibition (% ± SD)	Statistics ^1^	F3 Inhibition (% ± SD)	Statistics ^2^	Self-Inhibition (% ± SD)	Statistics ^1^	F3 Inhibition (% ± SD)	Statistics ^2^
T002	*T. harzianum*	93.18 ± 1.21	A	99.51 ± 0.56	a	95.04 ± 0.87	A	95.25 ± 1.89	a
T007	*T. harzianum*	91.68 ± 1.89	A,B	98.54 ± 1.26	a	89.87 ± 2.67	A,B	92.93 ± 2.16	a,b
T008	*T. citrinoviride*	74.51 ± 1.66	D	70.73 ± 1.38	e	71.02 ± 1.85	C	59.38 ± 2.93	d
T015	*T. harzianum*	93.86 ± 1.60	A	97.07 ± 1.59	a,b	94.62 ± 1.21	A	89.40 ± 2.28	b
T021	*T. harzianum*	91.43 ± 3.77	A,B	99.02 ± 0.00	a	90.08 ± 4.25	A,B	92.38 ± 0.91	a,b
T028	*T. velutinum*	42.29 ± 1.40	E	34.15 ± 3.03	f	23.57 ± 2.12	D	15.89 ± 1.96	e
T044	*T. harzianum*	90.09 ± 1.79	A,B	91.35 ± 2.55	c,d	89.66 ± 1.71	A,B	92.93 ± 3.22	a,b
T050	*T. harzianum*	91.11 ± 1.48	A,B	88.91 ± 0.51	c,d	89.98 ± 0.94	A,B	93.46 ± 0.42	a,b
T055	*T. harzianum*	87.86 ± 0.97	B	86.92 ± 1.12	d	87.66 ± 1.35	B	90.40 ± 1.11	a,b
T057	*T. gamsii*	82.53 ± 2.91	C	92.90 ± 4.86	b,c	68.74 ± 3.65	C	76.27 ± 2.02	c

^1^ Different capital letters indicate significant differences in self-inhibitory activity among the Trichoderma isolates (*p* ≤ 0.05). ^2^ Different lowercase letters indicate significant differences in antifungal activity against F3 among the Trichoderma isolates (*p* ≤ 0.05). PIs were estimated in relation to the control using the following equation: PI (%) = [(C − T/C) × 100] [16]. A one-way analysis of variance (ANOVA, *p* ≤ 0.05) was performed and differences were estimated using Tukey’s post hoc test (*p* ≤ 0.05).
